# Supplementary material for: Antimicrobial Resistance and Virulence Characterization of Listeria monocytogenes Strains Isolated from Food and Food Processing Environments
Source: Pathogens. 2022 Sep 25;11(10):1099. doi: 10.3390/pathogens11101099 (PMC9607609; doi:10.3390/pathogens11101099)
Supplement: Supplementary file 1 [file pathogens-11-01099-s001.zip › pathogens-1909954-supplementary.pdf]

Table S1. Antimicrobial resistance and virulence characteristic of all strains used in the study.

No.	Strain	Identification		Serotype	Isolation source	Biofilm	Slime production	Virulence-associated genes		Phenotypic antibiotic resistance	Minimum inhibitory concentration (MIC) (μg/ml)	Antibiotic-resistance genes
		PCR	MALDI-TOF MS					LIPI-1	Biofilm			
					**Strains from food**							
**1.**	116	*L. monocytognes*		1/2a	Farm cheese	No biofilm	No	*hlyA, prfA*	*inlB*	DA (I)	DA – 1	*lnuA*
**2.**	130	*L. monocytognes*		1/2a	Farm cheese	No biofilm	No	*hlyA, prfA*	*inlB*	DA (I)	DA – 1.5	*lnuA*
**3.**	137	*L. monocytognes*		1/2a	Raw milk	Moderate	No	*hlyA, prfA*	*inlB*	DA (R)	DA – 4	-
**4.**	138	*L. monocytognes*		1/2a	Raw milk	Moderate	No	*hlyA, prfA*	*inlB*	DA (R)	DA – 4	-
**5.**	140	*L. monocytognes*		1/2a	Raw milk	Weak	No	*hlyA, prfA*	*inlB*	DA (R)	DA – 4	-
**6.**	141	*L. monocytognes*		1/2a	Frozen vegetables	No biofilm	No	*hlyA, prfA*	*inlB, luxS, sigB*	DA (I)	DA – 2	*mefA, lnuA*
**7.**	142	*L. monocytognes*		1/2a	Frozen vegetables	No biofilm	No	*hlyA, prfA*	*inlB, luxS, sigB*	DA (I)	DA – 1	*lnuA*
**8.**	147	*L. monocytognes*		1/2a	Dumplings	No biofilm	No	*hlyA, prfA*	*inlB, luxS, sigB*	DA (I)	DA – 1.5	*mefA, lnuA*
**9.**	148	*L. monocytognes*		1/2a	Frozen vegetables	No biofilm	No	*hlyA, prfA*	*inlB, luxS, sigB*	CN (I), SXT (R)	CN – 0.19, SXT – 0.064	*aadB, mefA, lnuA, sulII*
**10.**	91	*L. monocytognes*		1/2a	Juice	No biofilm	No	*hlyA, prfA*	*inlB, luxS, sigB*	-	NA	NA
**11.**	92	*L. monocytognes*		1/2a	Juice	No biofilm	No	*hlyA, prfA*	*inlB, luxS, sigB*	DA (R), MEM (R), SXT (R)	DA – 2, MEM – 0.047, SXT – 0.064	*sulI*
**12.**	93	*L. monocytognes*		1/2a	Frozen vegetable	No biofilm	Yes	*hlyA, prfA*	*inlB, luxS, sigB*	DA (R)	DA – 32	*lnuA*
**13.**	94	*L. monocytognes*		1/2a	Frozen vegetable	No biofilm	No	*hlyA, prfA*	*inlB, luxS, sigB*	DA (I)	DA – 1	-
**14.**	95	*L. monocytognes*		1/2a	Frozen vegetable	No biofilm	No	*hlyA, prfA*	*inlB, luxS, sigB*	DA (I)	DA – 1.5	-
**15.**	96	*L. monocytognes*		1/2a	Chicken wings	No biofilm	Yes	*hlyA, prfA*	*inlB, luxS, sigB*	-	NA	NA
**16.**	98	*L. monocytognes*		1/2a	Farm cheese	No biofilm	Yes	*hlyA, prfA*	*inlB, luxS, sigB*	CIP (I), DA (I)	CIP – 0.38, DA – 1.5	*Ide*
**17.**	99	*L. monocytognes*		1/2a	Farm cheese	Moderate	No	*hlyA, prfA*	*inlB, luxS, sigB*	DA (I)	DA – 1	*mefA*
**18.**	139	*L. monocytognes*		1/2a	Raw milk	Moderate	No	*hlyA, prfA*	*inlB, luxS, sigB*	DA (R)	DA – 4	-
**19.**	117	*L. monocytognes*		1/2c	Raw milk	No biofilm	No	*hlyA, prfA*	*inlB*	-	NA	NA
**20.**	129	*L. monocytognes*		1/2c	Raw milk	No biofilm	No	*hlyA, prfA*	*luxS*	DA (I)	DA – 1.5	-
**21.**	146	*L. monocytognes*		3c	Frozen vegetables	No biofilm	No	*hlyA, prfA*	*inlB, sigB*	CIP (I), DA (I)	CIP – 0.50, DA – 1.5	*Ide, mefA, lnuA*
**22.**	149	*L. monocytognes*		3c	Frozen vegetables	No biofilm	No	*hlyA, prfA*	*inlB, luxS, sigB*	DA (I)	DA – 2	*mefA, lnuA*
**23.**	81	*L. monocytognes*		3c	Frozen vegetable	Strong	No	*hlyA, prfA*	*inlB, luxS, sigB*	DA (R)	DA – 4	-
**24.**	97	*L. monocytognes*		3c	Chicken breast fillet	No biofilm	Yes	*hlyA, prfA*	*inlB, luxS, sigB*	DA (R)	DA – 1.5	-
**25.**	143	*L. monocytognes*		Other	Frozen vegetable	No biofilm	No	*hlyA, prfA*	*inlB, luxS, sigB*	DA (R)	DA – 4	*lnuA*
**26.**	144	*L. monocytognes*		Other	Frozen vegetable	Strong	No	*hlyA, prfA*	*inlB, luxS, sigB*	DA (I)	DA – 2	*mefA, lnuA*
**27.**	145	*L. monocytognes*		Other	Frozen vegetable	No biofilm	No	*hlyA, prfA*	*inlB, luxS, sigB*	DA (I)	DA – 2	*mefA, lnuA*
					**Strains from food processing environments**							
					Food processing envionments							
**1.**	172	*L. monocytognes*		1/2a	Floor drain	Weak	No	*hlyA, prfA*	*inlB, luxS, sigB*	DA (I)	DA – 1.5	-
**2.**	167	*L. monocytognes*		1/2a	Floor drain	Moderate	No	*hlyA, prfA*	*inlB, luxS, sigB*	DA (I)	DA – 1.5	-
**3.**	165	*L. monocytognes*		1/2c	Floor drain	Weak	No	*hlyA, prfA*	*inlB, luxS, sigB*	P (R), SXT (R)	P – 1, SXT – 0.125	*mefA, sulI*
**4.**	168	*L. monocytognes*		1/2c	Floor drain	Strong	No	*hlyA, prfA*	*inlB, luxS, sigB*	DA (I)	DA - 2	*lnuA*
**5.**	169	*L. monocytognes*		1/2c	Venting system	No biofilm	No	*hlyA, prfA*	*inlB, luxS, sigB*	DA (R)	DA – 4	-
**6.**	170	*L. monocytognes*		1/2c	Production machine	Moderate	No	*hlyA, prfA*	*inlB, luxS, sigB*	DA (R)	DA – 4	-
**7.**	174	*L. monocytognes*		1/2c	Production machine	Weak	No	*hlyA, prfA*	*inlB, luxS, sigB*	DA (I), SXT (R)	DA – 1.5, SXT – 0.125	*sulI*
**8.**	176	*L. monocytognes*		1/2c	Production machine	No biofilm	No	*hlyA, prfA*	*inlB, luxS, sigB*	DA (I)	DA – 1.5	-
**9.**	177	*L. monocytognes*		1/2c	Production line	Weak	No	*hlyA, prfA*	*inlB, luxS, sigB*	DA (I), SXT (R)	DA – 1, SXT – 0.064	*sulI, sulII*
**10.**	164	*L. monocytognes*		3a	Production line	No biofilm	No	*hlyA, prfA*	*inlB, luxS, sigB*	DA (I)	DA – 2	-
**11.**	173	*L. monocytognes*		3a	Production line	Moderate	No	*hlyA, prfA*	*inlB, luxS, sigB*	DA (I)	DA – 2	-
**12.**	166	*L. monocytognes*		3c	Production line	Moderate	No	*hlyA, prfA*	*inlB, luxS, sigB*	DA (R)	DA – 4	-
**13.**	171	*L. monocytognes*		3c	Production line	Weak	No	*hlyA, prfA*	*inlB, luxS, sigB*	DA (I)	DA – 2	-

I – Intermediate, R – Resistance; NA – not applicable; AMP – Ampicillin, C – Chloramphenicol, CIP – Ciprofloxacin, E – Erythromycin, CN – Gentamicin, DA – Clindamycin, MEM – Meropenem, P – Penicillin G, RD – Rifampicin, SXT – Trimethoprim/Sulfamethoxazole, TE – Tetracycline, VA – Vancomycin.

Table S2. Primer sequence, product size, PCR protocol, and references used for the detection of antimicrobial resistance genes in *L. monocytogenes*.

Antimicrobial agent	Target Genes	Primer Sequence (5′–3′)	Product Size (bp)	Concentration	PCR cycling condition	Reference
**CIP**	*Lde*	F: ATCGTGAACTTAATGGTGG<br>R: ATCCTCATATAACTCAAGCG	1518	0.2 µM	Initial denaturation for 3 min at 95 °C, followed by 1 min of denaturation at 95 °C, 45 s annealing at 45 °C, 1 min of extension at 72 °C for a total of 35 cycles and 5 min of final extension at 72 °C.	[47]
**DA**	*lnuA*	F: GGTGGCTGGGGGGTAGATGTATTAACTGG<br>R: GCTTCTTTTGAAATACATGGTATTTTTCGATC	323	0.4 µM	Initial denaturation for 5 min at 94 °C, followed by 1 min of denaturation at 94 °C, 1 min annealing at 59 °C, 2 min of extension at 72 °C for a total of 35 cycles and 5 min of final extension at 72 °C.	[56]
	lnuB	F: CCTACCTATTGTTTGTGGAA<br>R: ATAACGTTACTCTCCTATTC	405	1 µM	Initial denaturation for 5 min at 94 °C, followed by 1 min of denaturation at 94 °C, 1 min annealing at 54 °C, 2 min of extension at 72 °C for a total of 35 cycles and 5 min of final extension at 72 °C.	
	mefA	F: AGTATCATTAATCACTAGTGC<br>R: TTCTTCTGGTACTAAAAGTGG	345	0.2 µM	Initial denaturation for 3 min at 95 °C, followed by 1 min of denaturation at 95 °C, 45 s annealing at 50 °C, 1 min of extension at 72 °C for a total of 35 cycles and 5 min of final extension at 72 °C.	[48]
**CN**	*aadB*	F: GAGCGAAATCTGCCGCTCTTG<br>R: CTGTTACAACGGACTGGCCGC	310	0.2 µM	Initial denaturation for 5 min at 95 °C, followed by 1 min of denaturation at 95 °C, 30 s annealing at 53 °C, 30 s of extension at 72 °C for a total of 40 cycles and 5 min of final extension at 72 °C.	[51]
	aac(3)-IIa(aacC2)[a]	F: CGGAAGGCAATAACGGAG<br>R: TCGAACAGGTAGCACTGAG	740	0.5 µM	Initial denaturation for 5 min at 94 °C, followed by 30 s of denaturation at 94 °C, 30 s annealing at 50 °C, 1,5 min of extension at 72 °C for a total of 30 cycles and 5 min of final extension at 72 °C.	[35]
**P**	*penA*	F: ATCGAACAGGCGACGATGTC<br>R: GATTAAGACGGTGTTTTACGG	500	0.2 µM	Initial denaturation for 3 min at 95 °C, followed by 1 min of denaturation at 95 °C, 45 s annealing at 46 °C, 1 min of extension at 72 °C for a total of 35 cycles and 5 min of final extension at 72 °C.	[51]
**SXT**	*sulI*	F: CGGCGTGGGCTACCTGAACG<br>R: GCCGATCGCGTGAAGTTCCG	433	0.2 µM	Initial denaturation for 5 min at 94 °C, followed by 30 s of denaturation at 94 °C, 30 s annealing at 65 °C, 2 min of extension at 72 °C for a total of 30 cycles and 10 min of final extension at 72 °C.	[32]
	sulII	F: GCGCTCAAGGCAGATGGCATT<br>R: GCGTTTGATACCGGCACCCGT	293	0.2 µM	Initial denaturation for 5 min at 94 °C, followed by 30 s of denaturation at 94 °C, 30 s annealing at 65 °C, 2 min of extension at 72 °C for a total of 30 cycles and 10 min of final extension at 72 °C.	

CIP – Ciprofloxacin, CN – Gentamicin, DA – Clindamycin, MEM – Meropenem, P – Penicillin G, SXT – Trimethoprim/Sulfamethoxazole.

Table S3. Primer sequence, product size, PCR protocol, and references used for identification, serotyping, and detection of virulence-associated genes in *L. monocytogenes* strains.

<table>
<tr><th colspan="2">Species/Gene/Serovar specificity</th><th>Primer Sequence (5′–3′)</th><th>Product Size (bp)</th><th>PCR cycling condition</th><th>Reference</th></tr>
<tr><td colspan="6">Identification</td></tr>
<tr><td colspan="2">Listeria genus
- prs</td><td>F: GCTGAAGAGATTGCGAAAGAAG
R: CAAAGAAACCTTGGATTTGCGG</td><td>370</td><td rowspan="2">Initial denaturation for 5 min at 94°C, followed by 30 s of denaturation at 94°C, 30 s annealing at 60°C, 30 s of extension at 72°C for a total of 35 cycles and 5 min of final extension at 72°C.</td><td rowspan="2">[9]</td></tr>
<tr><td colspan="2">L. monocytogenes
- lmo1030</td><td>F:GCTTGTATTCACTTGGATTTGTCTGG
R: ACCATCCGCATATCTCAGCCAACT</td><td>509</td></tr>
<tr><td colspan="6">Serotyping</td></tr>
<tr><td colspan="2">Serotype 1/2a and some serotype 3a strains
- flaA (lm_0690)</td><td>F: TTACTAGATCAAACTGCTCC
R: AAGAAAAGCCCCCTCGTCC</td><td>538</td><td rowspan="3">Initial denaturation for 3 min at 94°C, followed by 30 s of denaturation at 94°C, 30 s annealing at 52°C, 1 min of extension at 72°C for a total of 30 cycles and 5 min of final extension at 72°C.</td><td rowspan="3">[10]</td></tr>
<tr><td colspan="2">Serotype 1/2c strains
- LMOSLCC2372_0308</td><td>F: ATGCAACATCAAGAGCAAGAA
R: TGGCATTTCTAAGGATGTCTCT</td><td>300</td></tr>
<tr><td colspan="2">Most serotype 3a strains
- LMLG_0742</td><td>F: TGAGTTTGCAGGAAAGAAGG
R: AACCGTGGTTGGAACTGTAA</td><td>388</td></tr>
<tr><td colspan="6">Virulence-associated genes</td></tr>
<tr><td rowspan="2">LIPI-1</td><td>hlyA</td><td>F: GCAGTTGCAAGCGCTTGGAGTGAA
R: GCAACGTATCCTCCAGAGTGATCG</td><td>456</td><td>Initial denaturation for 3 min at 94°C, followed by 30 s of denaturation at 94°C, 30 s annealing at 55°C, 1 min of extension at 72°C for a total of 35 cycles and 5 min of final extension at 72°C.</td><td rowspan="5">[14]</td></tr>
<tr><td>prfA</td><td>F: GATACAGAAACATCGGTTGGC
R: GTGTAATCTTGATGCCATCAGG</td><td>274</td><td rowspan="3">Initial denaturation for 3 min at 94°C, followed by 30 s of denaturation at 94°C, 30 s annealing at 49°C, 1 min of extension at 72°C for a total of 35 cycles and 5 min of final extension at 72°C.</td></tr>
<tr><td rowspan="3">Biofilm</td><td>inlB</td><td>F: AAAGCACGATTTCATGGGAG
R: ACATAGCCTTGTTTGGTCGG</td><td>148</td></tr>
<tr><td>luxS</td><td>F: ATGGCAGAAAAAATGAATGTAGAAA
R: TTATTCACCAAACACATTTTTCCA</td><td>500</td></tr>
<tr><td>sigB</td><td>F: TCATCGGTGTCACGGAAGAA
R: TGACGTTGGATTCTAGACAC</td><td>310</td><td>Initial denaturation for 3 min at 94°C, followed by 30 s of denaturation at 94°C, 30 s annealing at 51°C, 1 min of extension at 72°C for a total of 35 cycles and 5 min of final extension at 72°C.</td></tr>
</table>
